# Disparities in Perpetrators, Locations, and Reports of Victimization for Sexual and Gender Minority Adolescents

**DOI:** 10.1016/j.jadohealth.2021.06.024

**Published:** 2021-08-23

**Authors:** Tessa M. L. Kaufman, Laura Baams

**Affiliations:** a Department of Sociology, University of Groningen, Groningen, The Netherlands; b Department of Education and Pedagogy, Utrecht University, Utrecht, The Netherlands; c Department of Pedagogy and Educational Sciences, University of Groningen, Groningen, The Netherlands

**Keywords:** Bullying, Victimization, Harassment, Sexual minority, LGBTQI+

## Abstract

**Purpose:**

Sexual and gender minority (SGM) adolescents are more likely to become victims of bullying and harassment than heterosexual, cisgender adolescents, but little is known about the contextual details of these victimization experiences. This study aims to examine by whom and where adolescents are bullied or harassed, to whom adolescents report such victimization, and whether these experiences differ between SGM and heterosexual, cisgender adolescents.

**Methods:**

Participants in this nationally representative study were 29,879 students (mean age = 14.1) from 136 Dutch middle/high schools across grades 7–12 (14.5% sexual minority, 2.7% gender minority) who completed a survey about their school-based experiences.

**Results:**

Perpetrators of victimization of SGM students were more often teachers and school staff compared with heterosexual, cisgender adolescents. Furthermore, SGM students experienced victimization in private locations (in the rest- or changing rooms/parking lots, at home), more often than heterosexual, cisgender students. Finally, SGM students felt less safe than their heterosexual, cisgender peers to report these experiences to teachers or parents, and were more likely to report their experiences to the police or the school janitor. SGM students who reported victimization experiences were less likely to receive support: the problems were less often acted on and persisted more often than those of heterosexual, cisgender students.

**Conclusions:**

SGM adolescents are not only victimized more often, but also by different perpetrators (teachers, other school staff) and in more private places. Their victimization is also less likely to be recognized or acted on by those responsible for adolescent's safety: teachers or parents.

Sexual and gender minority (SGM) adolescents are more likely to become victims of bullying and harassment than heterosexual, cisgender adolescents [1] and more often experience a poor school climate [2,3]. These stressors are thought to stem from hetero- and cisgender-normative social structures in which adolescents who deviate from these norms are considered to be inferior to the majority or dominant group of heterosexual, cisgender adolescents [4]. In the U.S., victimization disparities have decreased but not disappeared [1,2], and still many SGM adolescents frequently hear homophobic remarks (60.7%) or negative remarks about gender expression (53.2%) at school [3]. These experiences with victimization have detrimental health consequences, including depression [5–7], suicidality [8], and substance abuse [9]. In the Netherlands, social acceptance of diversity is relatively high [5,10], and all middle and high schools are mandated to cover sexual and gender diversity in their citizenship and civic skills curriculum and to have a general anti-bullying policy. Nevertheless, Dutch adolescents also experience SGM victimization [11] and health disparities can be observed, even after increased social acceptance of sexual and gender diversity [12].

Targeting strategies and policies to prevent victimization of SGM adolescents and detrimental health outcomes requires detailed knowledge on the context of adolescents' victimization experiences: who the perpetrators are, where victimization occurs, and reporting behaviors. However, little is known about these contextual details of SGM victimization, and the few existing studies focused on either the general population [13–16] or on SGM adolescents only [3], while comparing SGM with heterosexual, cisgender adolescents is crucial to a comprehensive understanding of SGM adolescents' unique experiences and needs.

## Perpetrators, locations, and reports of victimization

Identifying the perpetrators of victimization is central in developing prevention efforts. Specifically, SGM adolescents are at risk of experiencing bullying and harassment by peers [17], and consequently, current school practices and programs aim to prevent or reduce *peer* victimization [1]. However, victimization—especially of SGM adolescents—may also be carried out by others than peers, including teachers and other adults. This might result from sociocultural norms and individual biases that are present in adolescents and adults alike [3,18–20]. For example, in a U.S.- based sample, more than half (52.4%) of SGM adolescents heard homophobic remarks from school staff, and two thirds (66.7%) heard negative remarks from staff about gender expression [3]. In addition to victimization, studies show that teachers regularly ignore victimization of SGM adolescents, and do not intervene when witnessing it [3,18,21,22]. As such, it is likely that those who hold the responsibility to protect students against victimization and health disparities [23] might be an overlooked group in current prevention and intervention efforts.

Furthermore, adjusting prevention strategies to the high-risk *locations* where SGM adolescents experience victimization is needed to effectively protect these adolescents. A general adolescent population-based study showed that the classroom, lunchroom, and hallway were places where victimization occurred most often, while restrooms were relatively “safe” [15]. However, SGM adolescent experiences may differ. For example, gender minority adolescents are often prohibited to use the bathroom or locker room of their gender, and if they are able to use the bathroom of their gender they might be at risk for victimization in those locations [3,24]. Thus, an important question is on which high-risk locations (within and beyond school) preventive efforts should focus. Furthermore, reporting bullying and harassment to school staff is a critical step to improve classroom climate and to prevent victimization. Although most adolescents are hesitant to report victimization, U.S.-based findings showed that SGM adolescents are even less likely to report victimization to school staff [3]. They often doubt that effective intervention would occur, fear that staff members may be homophobic, or that reporting would make the situation worse [3]. In addition, a Chilean study reported that SGM adolescents were more likely to distrust school staff [18]. Moreover, SGM adolescents are also unlikely to report victimization to their parents [3].

In sum, little is known about the contextual details of victimization among SGM compared to heterosexual, cisgender adolescents that are crucial to effectively target interventions. Therefore, this study aimed to examine by whom and where SGM adolescents are victimized, whether and to whom SGM adolescents report victimization, and whether these experiences differ from those among heterosexual, cisgender adolescents. In doing so, we mostly focus on the school context as this is the place where adolescents spend most of their time.

## Methods

### Participants and procedures

The data from this study came from the nationally representative Social Safety Monitor, a school-based study commissioned by the Dutch Ministry of Education, Culture, and Science. All schools in the Netherlands were approached to participate in the study biennially. Schools were asked to administer the survey to students individually in classrooms behind separate desks. This research was exempt from Internal Review Board permission because the data collection was legally required as part of the law to monitor social safety in school, and commissioned by the Ministry of Education, Culture, and Science. Participation was voluntary, and data collection was anonymous; no individual login codes were used.

In total, 136 middle and high schools participated with adolescents across grades 7–12 (mean age = 14.12, standard deviation = 1.50). Data stem from two cohorts (2016 and 2018). Twenty percent of the schools participated in both cohorts and some students (except those who started attending middle school in 2017 or 2018, or finished high school after 2016) may therefore have participated twice, but due to the anonymous nature of the study we are unable to track individual students. However, school participation in both cohorts was not significantly related to levels of bullying, harassment, sexual orientation, gender identity, sex, or migration background. The final sample included 29,879 adolescents including heterosexual (N = 22,512; 85.5%), sexual minority ([SM]: N = 3,826; 14.5%), cis-gender (N = 27,369; 97.3%), and gender minority adolescents ([GM]: N = 766; 2.7%).

### Measures

Sexual orientation was assessed with the items “I could fall in love with a girl” and “I could fall in love with a boy” (1 = completely agree; 5 = completely disagree), in line with prior research [25,26]. We created a dummy variable representing a heterosexual orientation (0 = exclusively other-sex attracted; *completely agree/agree* with the statement about falling in love with another sex; and *completely disagree/disagree* with the statement about falling in love with the same sex) and SM orientation (1 = both-sex or same-sex attracted; *completely agree/agree* with the statement about falling in love with the same sex). Due to the sample size, we were unable to reliably test disparities for both-sex and same-sex attracted students separately. It was unknown whether students disclosed their sexual orientation to peers.

Gender identity was assessed as the extent to which students identified as a boy or a girl. Students were asked “Do you identify as a boy?” and “Do you identify as a girl?” (answer options: 0 = yes, completely, 1 = partly, 2 = no). By combining students' reported sex and gender identity [27], we created a dummy variable representing cisgender (0 = gender identity *completely* aligned with reported sex) versus GM (1 = gender identity *partly* or *not* aligned with the reported sex).

Perpetrators, locations, and reports of victimization were assessed among students who had experienced victimization. First, students were asked whether they were victimized, thus bullied or harassed, 58 months prior to the survey: that is, whether they were bullied (1 item: “how often have you been bullied since the summer holidays”) and whether they were physically harassed (mean of 11 items, sample item: “you were kicked on purpose”, α = .91). Answer options for both measures were 0 = never, 1 = less than once per month, 2 = once per month or more often, 3 = twice per week or more often, 4 = once per day or more often. Students who experienced either victimization or harassment (score >0) were asked about the perpetrators and locations of victimization and about their reporting behaviors: students could select multiple of the provided answer options for each question (e.g., they could select both “peers” and “teachers” as perpetrators), 0 = no, 1 = yes. All items and answer categories are shown in the tables in the Results section.

Covariates were levels of bullying/harassment victimization, sex ("What is your sex?," 0 = boy, 1 = girl), self-reported grade, educational level (0 = practical training and special education, 1 = prevocational education/pre-[applied] university education), and migration background (student's and parents' country of birth: 0 = both parents born in the Netherlands; 1 = at least one parent born outside the Netherlands).

### Analytical strategy

Disparities for SGM students were assessed with logistic regression analyses in Stata 16.0 (adjusted for clustering in schools using the *svy*: function). We first examined differences in bullying victimization and harassment for SGM students, respectively. SM status and GM status were used as independent variables in all analyses, and perpetrators of victimization, locations of victimization and perceived safety, and reporting behaviors were used as dependent variables.

Covariates were included in all analyses: sex, grade, educational level, and migration background. We also controlled for the school's participation in both cohorts. Furthermore, to eliminate confounding effects of the severity of the experiences on disparities we controlled for the average frequency of bullying victimization/harassment. Finally, for perpetrators and locations, we controlled for the other items of the same category because they were conceptually interrelated.

We conducted sensitivity analyses to check whether results for SM students were consistent when excluding both-sex attracted students, and when excluding students in schools that participated in both cohorts (excluded *N* = 9,133). For both sensitivity analyses, conclusions were similar (see Supplementary Information 1).

## Results

### Descriptive statistics

SGM students were more likely to experience bullying victimization and harassment, compared with heterosexual, cisgender students (Table 3, nonoverlapping confidence intervals). Of all groups, having experienced victimization through bullying and harassment were most common among GM and SM students compared to heterosexual, cisgender students. There were more female SGM than heterosexual, cisgender students, and GM students were relatively more likely than others to report a migration background and more likely to have a lower educational level. There were no age differences.

### Perpetrators of bullying and harassment

Different perpetrators of bullying and harassment were associated with SGM status (proportions in Table 1; adjusted odds ratios [AORs] in Table 4). Being bullied by peers was experienced more often by heterosexual, cisgender students than by SGM students, while being bullied by teachers (only for SM students), other school staff, or peers' family was more often reported by SGM students than by heterosexual, cisgender students. Furthermore, being harassed by other school staff or by peers' family was also more often reported by SGM students.

### Locations where bullying and harassment occurred

SGM status was also associated with the different locations where bullying and harassment took place (proportions in Table 1; AORs in Table 4). Being bullied or harassed took place less often in the classroom, but more often in restrooms or at home/perpetrator's home for SM than heterosexual students. Furthermore, bullying also occurred more frequently in the parking lot, and harassment occurred more often in the hallways/canteen for SM than heterosexual students. GM students were more likely than cisgender peers to be bullied in the changing rooms or the parking lot and to be harassed in restrooms. Finally, SGM students were more likely than heterosexual, cisgender peers to be bullied at home, at the perpetrator's home, or online, and SM students were also more likely to be harassed at home than heterosexual students.

### Reporting experiences of bullying and harassment

SGM status was associated with reporting behaviors (proportions in Table 2; AORs in Table 5). SM students were less likely than heterosexual students to report experiences of being bullied or harassed to parents, but more likely to report them to the police or report about being harassed to the school janitor. SM students were more likely than heterosexual students to not report harassment because they did not dare to, or to not report bullying or harassment because they did not know who to tell. For those who reported victimization, SM students more frequently felt that no action was taken to tackle bullying, and that harassment continued. Finally, SM students felt less able to count on their parents when bullied than heterosexual students.

GM students reported experiences of being bullied less often to parents than cisgender students, while reported bullying to a school janitor or (also about harassment) to the police more often. GM students were more likely to not dare to report that they were bullied or harassed than cisgender students, and more likely to feel that it was needed to report being bullied. For those who reported being bullied or harassed, the problems remained more frequently unsolved, or no action was taken to tackle bullying for GM than for cisgender students. Finally, GM students felt less able to count on their mentor or parents when being bullied than cisgender students.

## Discussion

Utilizing a representative school-based sample from middle and high schools in the Netherlands, the current study examined disparities in the context of victimization experiences for SGM and heterosexual, cisgender adolescents. Despite high social acceptance of diversity in the Netherlands [5,10], we found that SGM adolescents are more likely to experience bullying victimization and harassment than heterosexual, cisgender adolescents. Moreover, mechanisms of bullying and harassment appeared comparable to those observed in other countries.

Importantly, this study showed that perpetrators, locations, and (responses to) reports are different for victimized SGM adolescents versus victimized heterosexual, cisgender adolescents, implying that existing social safety practices and policies may be less successful for SGM adolescents. Specifically, our findings showed that, compared with heterosexual, cisgender adolescents, SGM adolescents were more likely to be bullied and harassed by teachers, other school staff, and peers' family members. Victimization occurred more often in private places such as restrooms, parking lots, at the adolescent's or perpetrator's home, or online. Furthermore, SGM adolescents were less likely than heterosexual, cisgender adolescents to report these victimization experiences because they did not know who to tell or because they did not dare to report it. They were also less likely to report the victimization experiences to their parents, but reported victimization to the police more often. For those who reported victimization, it was less likely that any action was taken or that the problem was solved for SGM victims than for heterosexual, cisgender victims.

Strikingly, our findings point to a group of perpetrators—teachers and other adults—that is often not included or targeted in (preventive) intervention strategies, but that has the responsibility to provide support and a safe school climate for all students. Furthermore, while troubling on its own, teachers' behaviors also send a message to adolescents that such behaviors are acceptable in the school [19], which then limit the effects of peer interventions or inclusive curricula [3,20]. These adults may hold more prejudice, or they might simply be unaware of the impact of their behaviors on SGM adolescents [28]. In addition, behavior of SGM adolescents is more often seen as norm-violating; SGM adolescents are often punished disproportionally for public displays of affection and self-expression, or for self-protection against victimization [29]. Whether these processes would explain perpetration of victimization by teachers and other school staff is currently unclear, and an important focus for future research.

Not only do our findings highlight that perpetrators of bullying and harassment also include teachers and other adults, but also that bullying and harassment of SGM adolescents occurs at different locations in and around the school or, in line with prior research, online [30]. In contrast to victimization literature on adolescents in general showing that bullying occurs most often in the classroom and hallways, and less often in restrooms [15], our findings showed that SGM adolescents were less likely to experience victimization in classrooms, but more likely to experience it in restrooms or changing rooms. These locations may be particularly unsafe for GM adolescents, as they are usually sex-segregated and GM adolescents may be unable to use those of their gender. In line, previous research showed that transgender adolescents reported more restroom unsafety than cisgender adolescents, and therefore reported lower school safety [31]. Furthermore, not only restrooms but also other private locations such as the parking lot, hallways, and online were higher risk locations for victimization of SGM adolescents compared to victimized heterosexual, cisgender peers, which generally suggests that unsupervised locations are a particular risky context for SGM adolescents. Importantly, our findings indicate that in the design of antibullying programs and interventions for middle and high schools, the focus should not just be on the classroom or other more public spaces, but should also target victimization that occurs in more private areas.

When considering perpetrators and locations of victimization, it is not surprising that SGM adolescents were more than twice as likely to not *dare to* report victimization, or to not *know* whom to report it to, compared with heterosexual, cisgender adolescents. Perhaps they fear further rejection, retaliation, or being outed to others [3]. Furthermore, even beyond the school context SGM adolescents reported their experiences with victimization to their parents less often than heterosexual, cis-gender adolescents, and felt that they could not count on their parents when being bullied. This aligns with research among U.S. adolescents [3] and with earlier research among Dutch adolescents [11], in which parents of victimized SM youth underestimated their child's problems. In contrast, SGM adolescents were more likely to report victimization to the police or the school janitor. In the Netherlands, the school janitor provides facility services, but also supervises students in detention, and students regularly encounter them in different school locations. Students might feel safe with them in the absence of a formal or dependent relationship, or might report victimization to them because they may witness victimization in more private places in the school.

In sum, SGM adolescents seem more hesitant about seeking social support from school staff or parents: perhaps because these people are the perpetrators, or because they may be afraid of outing themselves to them or might fear rejection. It is therefore vital that schools do not only focus on creating a safe and inclusive school climate, but also provide clear information to adolescents about how they can access supportive and potentially anonymous resources that can help them when they experience victimization. Furthermore, to decrease victimization disparities and improve SGM adolescents' well-being, schools can use Lesbian, Gay, Bisexual, Transgender, and Questioning-inclusive sex education [32] or more specific student-directed initiatives, known as Gender Sexuality Alliances [33,34]. Finally, it is vital that inclusion and diversity are mandatory topics in school staff's training curriculum.

### Strengths and limitations

This study went beyond identifying general disparities in victimization for SGM adolescents, and zoomed in on contextual details of these experiences that are crucial to improve strategies and target interventions, and ultimately decrease the harmful peer experiences that many SGM adolescents have. Moreover, this is the first study on these contextual details of victimization that includes both SGM and heterosexual, cisgender populations, which enables comparisons between the experiences and needs of both populations. Finally, it is also the first nationally representative study on SGM adolescents' experiences in the Netherlands. Despite these strengths, this study has several limitations. First, there may be within-group SGM differences or gender [35] or age differences in our findings, but our sample of victimized SGM adolescents was too small to reliably test these subgroup differences and our measures of sexual and gender identity did not adequately capture sexual orientation or gender identity/expression subgroups. This lack of comprehensive measures is a common problem in national surveys and we recommend future projects address this issue [27,36]. Second, we only included adolescents' self-reports. Although victimization in private locations such as restrooms or the home environment is difficult to observe by peers or teachers, they could be helpful informants to understand disparities in the recognition of victimization. Finally, we did not have any health indicators and could therefore not estimate associations between victimization disparities and health outcomes. However, these associations have been repeatedly demonstrated in previous research [5–8,37], highlighting the importance of our findings for the health domain.

In conclusion, although the Netherlands is a country with relatively high social acceptance of sexual and gender diversity [5,10], the current findings indicate important disparities in school-based experiences for SGM adolescents. This study adds to a large body of research showing that adolescence is a particularly vulnerable period for SGM adolescents. Our study showed that SGM adolescents are more likely to experience victimization, and that the perpetrators are, compared to heterosexual, cisgender youth more often those who are also responsible for their safety and wellbeing: teachers, parents, and other adults. Moreover, SGM adolescents are more likely to be victimized in private locations and to feel less safe to report these experiences; and when they do report them, they are less likely to receive support. Future studies could examine adolescent-, teacher-, and school-related factors that explain different recognition and response patterns of SGM adolescents' victimization and how these factors affect adolescent mental health. School policies need to focus on increasing the safety of not only the public but also more private areas, and on providing adolescents with information about supportive and potentially anonymous resources or school personnel [38], which can help to improve the health of all adolescents.

## Supplementary Material

Supplementary data related to this article can be found at https://doi.org/10.1016/j.jadohealth.2021.06.024.

Supplementary Material

## Figures and Tables

**Table 1:** Adjusted proportions of perpetrators and locations of bullying and harassment across sexual and gender identity

	Bullying				Harassment				Bullying				Harassment			
	Heterosexual		Sexual minority		Heterosexual		Sexual minority		Cisgender		Gender minority		Cisgender		Gender minority	
	%	95% CI	%	95% CI	%	95% CI	%	95% CI	%	95% CI	%	95% CI	%	95% CI	%	95% CI
Perpetrators: Who were the perpetrators of victimization?																
Peers	92.8	[91.3–94.0]	88.4	[84.7–91.3]	59.1	[57.3–61.0]	64.4	[60.5–68.2]	92.7	[91.1–94.0]	86.7	[82.0–90.3]	60.0	[58.1–61.9]	70.5	[64.1–76.2]
Teachers	13.6	[11.5–16.0]	23.7	[19.7–28.2]	7.6	[6.6–8.6]	15.1	[12.6–17.9]	12.8	[10.9–14.9]	43.1	[35.6–50.9]	8.0	[7.2–8.9]	27.6	[23.0–32.9]
Other school staff	8.4	[6.8–10.2]	20.3	[16.1–25.3]	4.4	[3.8–5.1]	12.7	[10.1–15.8]	8.8	[7.4–10.4]	34.0	[27.1–41.7]	4.8	[4.1–5.6]	25.2	[20.2–30.8]
Peers' family	14.3	[12.6–16.1]	25.2	[20.3–30.9]	6.8	[6.0–7.7]	15.3	[12.8–18.2]	14.4	[12.8–16.1]	44.1	[36.5–52.0]	7.5	[6.7–8.3]	27.3	[22.6–32.6]
Locations: Where did victimization take place?																
School																
Classroom	75.8	[73.0–78.4]	72.0	[66.9–76.6]	40.1	[38.0–42.2]	44.3	[40.9–47.8]	74.9	[72.3–77.2]	75.0	[68.2–80.8]	40.6.	[38.6–42.6]	56.2	[50.6–61.7]
Study/work space	38.0	[34.3–41.8]	45.5	[40.6–50.5]	17.0	[15.3–18.9]	26.3	[23.3–29.6]	38.1	[34.8–41.6]	54.3	[46.9–61.6]	17.9	[16.3–19.7]	35.7	[30.1,41.7]
Hallways, canteen	76.4	[73.8–78.8]	77.0	[72.3–81.1]	51.7	[49.5–53.9]	61.1	[58.1–64.2]	76.2	[73.6–78.6]	79.9	[73.8–84.8]	52.8	[50.9–54.8]	65.8	[59.7–71.5]
Restrooms	16.0	[14.2–18.0]	25.9	[21.6–30.6]	8.7	[7.5–10.1]	20.5	[17.7–23.7]	16.0	[14.3–17.7]	35.3	[27.2–44.5]	9.5	[8.2–10.8]	32.9	[27.7–38.6]
Changing rooms	35.2	[32.3–38.2]	40.9	[36.5–45.6]	15.8	[14.0–17.7]	24.1	[21.0–27.4]	35.6	[32.8–38.3]	51.1	[43.5–58.6]	16.6	[14.9–18.5]	35.4	[30.6–40.5]
Schoolyard or around school	60.6	[57.6–63.5]	66.6	[62.4–70.6]	36.2	[33.4–39.1]	46.3	[42.2–50.6]	61.7	[58.6–64.7]	70.7	[63.7–76.8]	37.7	[34.9–40.6]	57.5	[51.7–63.1]
Parking lot	32.9	[30.3–35.4]	41.4	[36.3–46.6]	18.6	[16.8–20.4]	31.0	[27.1–35.0]	32.3	[30.0–34.8]	54.3	[46.2–62.2]	19.8	[18.0–21.8]	40.1	[35.1–45.2]
Home																
At home	14.8	[12.9–16.9]	28.0	[23.8–32.7]	8.7	[8.0–9.5]	18.9	[16.2–21.8]	16.3	[14.5–18.2]	38.0	[29.2–47.7]	9.4	[8.7–10.2]	29.5	[24.2–35.4]
At the perpetrators' home	8.0	[6.6–9.5]	17.0	[13.0–22.0]	6.8	[5.9–7.8]	15.8	[13.2–18.7]	8.0	[6.9–9.2]	33.7	[25.5–43.0]	7.3	[6.5–8.2]	27.3	[21.7–33.8]
Online	39.7	[39.9–42.5]	48.0	[43.8–52.1]					40.5	[38.3–42.8]	55.1	[47.1–62.8]				

Percentages reflect proportion scores transformed into percentages, adjusted for clustering at the school level. CI = confidence interval.

**Table 2:** Adjusted proportions of reports of bullying and harassment across sexual and gender identity

	Bullying				Harassment				Bullying				Harassment			
	Heterosexual		Sexual minority		Heterosexual		Sexual minority		Cisgender		Gender minority		Cisgender		Gender minority	
	%	95% CI	%	95% CI	%	95% CI	%	95% CI	%	95% CI	%	95% CI	%	95% CI	%	95% CI
To whom did you report victimization?																
Peers	25.0	[22.7–27.4]	27.6	[23.3–32.3]	18.0	[16.8–19.2]	22.2	[19.9–24.8]	26.0	[24.0–28.1]	26.8	[21.6–32.9]	19.0	[17.8–20.2]	22.6	[18.3–27.6]
Parents	59.2	[56.5–61.8]	50.4	[45.4–55.4]	28.0	[25.7–30.5]	29.0	[25.5–32.8]	58.9	[56.2–61.5]	40.5	[33.2–48.3]	28.7	[26.5–30.9]	29.7	[24.1–35.9]
Mentor	49.1	[45.6–52.6]	47.9	[43.3–52.5]	18.8	[16.4–21.5]	23.2	[19.9–26.9]	51.1	[47.7–54.4]	41.6	[34.3–49.0]	20.0	[17.6–22.6]	26.9	[22.0–32.5]
School janitor	5.1	[4.0–6.5]	6.7	[4.5–9.8]	2.1	[1.7–2.7]	5.1	[3.9–6.6]	5.0	[4.0–6.4]	12.1	[7.8–18.2]	2.5	[2.0–3.1]	7.3	[4.8–11.0]
School counselor	16.0	[13.7–19.6]	19.0	[15.0–23.9]	5.5	[4.6–5.9]	8.4	[6.7–10.5]	16.4	[14.5–18.6]	19.5	[13.5–27.2]	5.8	[4.9–6.8]	11.3	[8.1–15.5]
Other staff	10.8	[9.1–12.8]	14	[11–18]	4.4	[3.6–5.5]	8.0	[6.3–10.1]	10.9	[9.4–12.7]	14.7	[10.3–20.6]	4.8	[4.0–5.7]	10.7	[7.8–14.4]
Police	3.9	[2.9–5.1]	7.9	[5.7–11.0]	2.3	[1.8–2.9]	6.4	[4.8–8.1]	3.9	[3.1–5.0]	13.2	[8.9–19.0]	2.5	[2.0–3.1]	11.6	[8.3–16.1]
Other	9.1	[7.5–10.8]	14.2	[11.4–17.6]	17.8	[16.6–19.0]	15.0	[12.8–17.5]	9.5	[8.3–10.9]	17.9	[13.6–23.2]	17.5	[16.3–18.7]	15.3	[11.8–19.6]
Why did you not report victimization?																
Didn't dare to	26.1	[21.3–31.4]	38.7	[28.3–50.3]	5.7	[4.7–6.8]	17.1	[13.2–21.8]	26.7	[22.5–31.3]	47.8	[33.9–62.1]	6.5	[5.4–7.8]	24.5	[17.7–32.8]
Didn't know to whom	5.7	[3.6–9.0]	14.0	[8.7–21.6]	3.3	[2.6–14.3]	8.4	[5.3–12.8]	6.5	[4.3–9.7]	17.4	[8.8–31.3]	3.9	[3.1–5.0]	8.5	[4.3–16.3]
Didn't seem needed	62.1	[56.3–67.5]	45.2	[34.1–56.8]	67.4	[64.8–69.9]	62.1	[57.6–66.5]	58.7	[53.8–63.4]	45.7	[32.1–59.9]	67.3	[64.9–69.6]	48.9	[39.1–58.8]
What happened after reporting?																
Problem is unsolved	30.5	[27.3–33.9]	26.9	[22.6–31.7]	12.3	[10.8–13.9]	17.7	[15.1–20.6]	29.7	[26.9–32.7]	35.5	[28.6–43.0]	13.5	[12.3–14.8]	20.4	[14.6–27.6]
No action was taken	21.5	[18.9–24.3]	30.9	[25.7–36.7]	40.4	[38.1–42.8]	40.1	[36.1–44.2]	22.6	[20.4–25.1]	34.0	[25.7–43.5]	39.5	[37.5–41.4]	42.1	[34.3–50.2]
I can count on this person when being bullied…																
Mentor	74.3	[70.9–77.4]	64.0	[57.3–70.1]					75.3	[72.5–77.9]	51.0	[42.4–59.4]				
School head	51.2	[47.3–55.1]	44.4	[39.1–49.9]					51.7	[48.4–55.2]	40.8	[32.6–49.5]				
School counselor	64.4	[60.7–68.0]	56.9	[50.4–63.2]					64.6	[61.3–67.7]	45.9	[37.8–54.2]				
Parents	81.6	[79.0–84.0]	68.9	[62.4–74.8]					81.9	[79.5–84.0]	59.2	[51.2–66.9]				

Percentages reflect proportion scores transformed into percentages, adjusted for clustering at the school level. CI = confidence interval.

**Table 3:** Estimated adjusted proportions of bullying and harassment victimization by sexual and gender minority status

	Heterosexual (N = 22,512)		Sexual minority (N = 3,826)		Cisgender (N = 27,369)		Gender minority (N = 766)	
	%	95% CI	%	95% CI	%	95% CI	%	95% CI
Bullying	13.0	[11.5–14.7]	21.9	[19.3–24.5]	13.8	[12.2–15.4]	35.6	[32.2–38.9]
Harassment	27.9	[26.4–29.5]	31.2	[29.1–33.4]	27.5	[25.9–29.0]	42.8	[38.8–47.0]
Sex assigned at birth	43.8	[40.8–46.9]	58.1	[55.8–60.3]	46.1	[43.3–49.0]	70.3	[66.1–74.2]
Migration background	18.8	[14.5–24.1]	22.5	[18.7–26.9]	19.6	[15.3–24.6]	37.9	[31.6–42.6]
Educational level	92.8	[88.7–95.4]	89.5	[83.8–93.4]	91.9	[87.5–94.8]	86.2	[79.4–91.0]
	M	SD	Mean	SD	Mean	SD	Mean	SD
Age	14.2	1.5	14.1	1.6	14.2	1.5	14.3	1.7

For sex: male = 0, female = 1; for migration background: 0 = no migration background, 1 = migration background; for educational level: 0 = practical training and special education. Percentages reflect proportion scores transformed into percentages, adjusted for clustering at the school level. CI = confidence interval; SD = standard deviation.

**Table 4:** Logistic regression results of associations between SGM status and perpetrators, locations and reports of bullying and harassment

	Heterosexual (0) versus sexual minority adolescents (1)				Cisgender (0) versus gender minority adolescents (1)			
	Bullying		Harassment		Bullying		Harassment	
	AOR	95% CI	AOR	95% CI	AOR	95% CI	AOR	95% CI
Perpetrators: Who were the perpetrators of victimization?								
Peers	**.75**	[.59–.95]	1.05	[.85–1.30]	**.62**	[.41–.93]	.96	[.65–1.40]
Teachers	**1.35**	[1.00–1.82]	.92	[.67–1.25]	1.28	[.85–1.96]	.93	[.56–1.54]
Other school staff	**1.47**	[1.01–2.12]	**1.75**	[1.17–2.64]	**1.07**	[.65–1.78]	**2.08**	[1.20–3.59]
Peers' family	**1.62**	[1.26–2.08]	**1.48**	[1.15–1.91]	**1.89**	[1.24–2.87]	**1.59**	[1.03–2.44]
Locations: Where did victimization take place?								
School								
Classroom	**.79**	[.63–.98]	**.75**	[.63–.90]	.78	[.55–1.09]	1.11	[.80–1.53]
Study/work space	1.25	[.99–1.58]	1.24	[.99–1.54]	1.34	[.93–1.93]	.96	[.65–1.42]
Hallways, canteen	1.03	[.86–1.24]	**1.19**	[1.01–1.40]	.82	[.57–1.18]	.73	[.49–1.08]
Restrooms	1.17	[1.00–1.64]	**1.57**	[1.17–2.10]	1.08	[.69–1.67]	**2.00**	[1.32–3.03]
Changing rooms	1.17	[.95–1.44]	1.09	[.87–1.35]	**1.66**	[1.09–2.52]	1.40	[.94–2.10]
Schoolyard or around school	1.02	[.85–1.21]	1.02	[.85–1.22]	.88	[.63–1.23]	1.30	[.90–1.87]
Parking lot	**1.31**	[1.04–1.65]	1.20	[.91–1.57]	**1.54**	[1.09–2.18]	.96	[.65–1.43]
Home								
At home	**1.67**	[1.33–2.11]	**1.53**	[1.18–1.99]	**1.76**	[1.21–2.55]	1.40	[.92–2.14]
At the perpetrators' home	**1.42**	[1.04–1.94]	1.39	[.95–2.04]	**2.35**	[1.60–3.44]	1.43	[.89–2.33]
Online	**1.22**	[1.01–1.46]			**1.52**	[1.12–2.05]		

Results of logistic regressions with sexual and gender identity as independent variables (covariates: severity of victimization, sex assigned at birth, migration background, grade, school participated in two cohorts, other answer categories of the same question). AORs in bold reflect significant group difference. For parsimony, answers to three categories (corridors, lockers, canteen) were merged because in the Netherlands, most of these places overlap and students’ answers correlated strongly (r’s = .50–.60). AOR = adjusted odds ratio; CI = confidence interval; SGM = sexual and gender minority.

**Table 5:** Logistic regression results of associations between SGM status and reports of bullying and harassment

	Heterosexual (0) versus sexual minority adolescents (1)				Cisgender (0) versus gender minority adolescents (1)			
	Bullying		Harassment		Bullying		Harassment	
	AOR	95% CI	AOR	95% CI	AOR	95% CI	AOR	95% CI
To whom did you report victimization?							
Peers	1.10	[.88–1.37]	1.14	[.96–1.34]	1.15	[.82–1.62]	.80	[.57–1.14]
Parents	**.68**	[.58–.79]	.77	[.66–.92]	**.58**	[.41–.83]	.74	[.52–1.06]
Mentor	1.06	[.86–1.32]	1.16	[.98–1.42]	.83	[.56–1.21]	1.26	[.87–1.84]
School janitor	1.05	[.62–1.80]	**1.59**	[1.06–2.45]	**2.61**	[1.39–4.93]	1.45	[.81–2.55]
School counselor	1.03	[.74–1.42]	1.02	[.75–1.38]	1.18	[.76–1.83]	1.11	[.63–1.94]
Other staff	1.11	[.77–1.55]	1.21	[.86–1.71]	.89	[.54–1.46]	1.35	[.72–2.53]
Police	**1.91**	[1.15–3.19]	**1.77**	[1.20–2.60]	**2.15**	[1.12–4.14]	**2.14**	[1.24–3.69]
Other	1.19	[.89–1.60]	.84	[.67–.06]	1.39	[.94–2.06]	.84	[.58–1.21]
Why did you not report victimization?							
Didn’t dare to	1.45	[.89–2.37]	**2.72**	[1.72–4.30]	**2.11**	[1.03–4.28]	**2.18**	[1.10–4.32]
Didn’t know to whom	**2.61**	[1.30–5.23]	**1.84**	[1.06–3.18]	1.96	[.79–4.86]	1.27	[.49–3.32]
Didn’t seem needed	.73	[.49–1.10]	1.00	[.76–1.29]	.67	[.40–1.13]	**.49**	[.31–.79]
What happened after reporting?							
Problem is unsolved	1.25	[.96–1.63]	**1.47**	[1.10–1.95]	**2.25**	[1.56–3.26]	**1.75**	[1.08–2.82]
No action was taken	**1.40**	[1.08–1.83]	1.13	[.92–1.40]	**1.89**	[1.22–2.93]	1.30	[.89–1.91]
I can count on this person when being bullied…							
Mentor	.81	[.61–1.07]			**.49**	[.32–.73]		
School head	1.22	[.92–1.62]			1.31	[.79–1.85]		
School counselor	.84	[.65–1.10]			.83	[.60–1.16]		
Parents	**.61**	[.47–.80]			**.55**	[.39–.78]		

Results of logistic regressions with sexual and gender identity as dependent variables (covariates: severity of victimization, sex assigned at birth, migration background, grade, school participated in two cohorts). AORs in bold reflect significant group difference. AOR = adjusted odds ratio; CI = confidence interval; SGM = sexual and gender minority.
