# Supplementary material for: Disparities in Perpetrators, Locations, and Reports of Victimization for Sexual and Gender Minority Adolescents
Source: J Adolesc Health. Author manuscript; Available in PMC 2022 Jun 4. (PMC7612809; doi:10.1016/j.jadohealth.2021.06.024)
Supplement: Supplementary Material [file EMS145486-supplement-Supplementary_Material.docx]

Supplementary Information 1: Sensitivity Analyses

Two sensitivity analyses were performed: one excluding both-sex attracted students, and one excluding students who attended schools that had participated in both cohorts.

**Excluding both-sex attracted students**

First, overall, findings were consistent when excluding both-sex attracted students from the analyses (exclusively same-sex attracted students: *N* = 3,509), in terms of significance and differences (differences in *AOR*  <.30). Minor exceptions were that several effects became marginally significant instead of significant: effects of fellow students as perpetrators of bullying (*AOR* = 0.77; 95% *CI* = 0.59; 1.00), effects of being bullied in the restrooms (*AOR* = 1.25, 95% *CI* = 0.96; 1.64) and effects of reporting to the school janitor about harassment (*AOR* = 1.54; 95% *CI* = 1.00; 2.38). Further, the effect of no action taken as response to bullying became non-significant (*AOR* = 1.26; 95% CI = 0.95;1.67).

**Excluding schools that participated in both cohorts**

When only running the analyses on the sample of students who attended schools that participated in only one cohort (*N* = 23,670, excluded were *N* = 9,133 students), overall conclusions were similar. Differences in effect sizes were small (difference in *AOR*  < .35).

First, the effect of being more likely to be bullied by teachers became marginal for SM students (*AOR* = 1.36; 95% *CI* [0.96; 1.96]), as well as the effect of being more likely to be harassed by peers’ family for GM students (*AOR* = 1.54; 95% *CI* [0.93; 2.55]). The effect of being more likely to be bullied by teachers became significant for GM students (*AOR* = 2.23; 95% *CI* [1.48; 3.43]), but the effect more likely to be harassed by peers’ family became non-significant (*AOR* = 1.44; 95% *CI* [0.82; 2.50]).

Second, there was no significant effect of being bullied in the classroom (*AOR* = 0.82, 95% *CI* [0.65; 1.04]), restrooms (*AOR* = 1.25, 95% *CI* [0.92; 1.70]), or of harassment in the hallways/canteen (*AOR* = 1.19, 95% *CI* [0.97; 1.45]) for SM students. Further, the effects of being more likely to experience online victimization became marginal for SM students but equal in size (*AOR* = 1.20, 95% *CI* [0.98, 1.48]). In addition, there was an additional effect for GM students of being less likely to be harassed in the hallways/canteen (*AOR* = 0.56, 95% *CI* [0.38; 0.84]).
 Third, the effect of SM students being more likely to not report victimization because they did not know who to tell became marginal (for bullying, *AOR* = 2.09, 95% *CI* [0.90, 4.83], for harassment, (*AOR* = 1.33, 95% *CI* [0.65; 2.75]) while for GM students this effect became significant for bullying, *AOR* = 2.95, 95% *CI* [1.03; 8.44]).

Last, the effects of being more likely that no action was being taken for GM students (*AOR* = 1.32, 95% *CI* [0.98; 1.78]), or that the problem remained unsolved (*AOR* = 1.45, 95% *CI* [0.80; 2.62]) after reporting harassment, became marginally/non-significant.
